# Completion of the Second Volume of the American Journal and Library of Dental Science

**Published:** 1842-06

**Authors:** 


*Completion of the Second Volume of the American Journal and Library of Dental Science.*—The present number completes the second volume of this publication, and the occasion seems to be one, which calls for a remark or two from those under whose editorial management it has been placed. Before the first number of the third volume shall have been issued, another meeting of the American Society of Dental Surgeons, will have taken place, and as the editors are elected from year to year by this association, we know not that our present relationship to the Journal will continue any longer, but we think we may be permitted to say, that the volume now completed, evidences a determination on the part of those under whose auspices it is published, to send forth a periodical that shall be creditable to themselves and valuable to the profession; indeed, the flattering commendations it has already received from many of the most distinguished of our professional brethren both in this country and Europe, assure us that it is appreciated. The favourable manner too, in which it has been regarded and spoken of by the medical press and profession throughout the country, bear decided and unequivocal testimony in favour of its value, and we would here take occasion to observe, that these evidences of approbation and friendly regard, have been to us, as they must have been to every member of the society, and all who feel any interest in the advance of that department of medicine, to the dissemination of a knowledge of which, its pages are exclusively devoted, a source of peculiar gratification. And, these tokens of approbation have been the more grateful as they have been spontaneous on the part of those who have given them. Of the medical periodicals which have noticed it in terms of high commendation, it gives us pleasure to mention the following: "*The Boston Medical and Surgical Journal;*" "*The American Journal of Medical Sciences;*" "*The Bulletin of Medical Science;*" "*The New York Medical Gazette;*" "*The New York Lancet,*" and "*The Maryland Medical and Surgical Journal.*" For the gratification of such of the friends of the "American Journal and Library of Dental Science," as might be glad to know the estimation in which it is held by its medical cotemporaries, we copy from three or four of those just mentioned, what their editors have said concerning it.

The Editor of "The Boston Medical and Surgical Journal," in noticing the appearance of the first number after it had become the property of the American Society of Dental Surgeons, says—

*American Journal and Library of Dental Science.*—With the commencement of the second volume of this unique periodical, published in Baltimore and New York, we feel it incumbent on us to speak of its character, its influence, and its claims. If it had been announced, a dozen years ago, that a journal was about being published, exclusively devoted to the interests of dentistry, very likely the most judicious amongst us would have spoken of the impossibility of sustaining it, even if there were materials for filling its pages. Such a journal, however, does exist, of ample dimensions, and generous in all its expressions towards men in other pursuits. It is distinctly scientific in its character—and thus it carries its own recommendation to those who might apprehend that such a work would be made the instrument of a cunning or ambitious individual, who would not only use it for his own personal influence, but also convert it into an engine of oppression towards those whom he might wish to keep out of sight. No such feeling has ever been even remotely manifested. The Journal breathes nothing but a fervent disposition to distribute knowledge, and raise the brotherhood to an equal level of scientific eminence and respectability. The result of its influence, together with that of the Dental College, on the character of the dental profession in the United States, must therefore be incalculably beneficial. It will also tend to purge the land of all pretenders to dentistry. The public sentiment accords most harmoniously with the efforts of the leading members of the new dental association. People will not have their teeth ruined by an ignoramus, because they can perceive his skill by the manner of operation, however willingly they may swallow boluses that sap the foundation of health. In short, this Journal forms an era in modern dentistry, of which those devoted to the business, may well be proud. Not to encourage this publication, now published under the auspices of the American Society of Dental Surgeons, is an evidence of being behind the age, and denotes wilful ignorance of the rapid improvements in that important department of science. Physicians and associations should order it for the two-fold object of personal improvement and encouragement of the worthy proprietors.

From "The American Journal of Medical Science," we copy the following—

"*The American Journal and Library of Dental Science.*—This work is at present edited by Chapin A. Harris, M. D. of Baltimore, and Solyman Brown, M. D. of New York, and is published under the auspices of the American Society of Dental Surgeons. Since the existing arrangement, which commenced with the second volume, (Oct. 1841,) the work has been greatly improved in its appearance. It is every way exceedingly creditable to all concerned, and one, we should think, indispensable to every dentist who desires to keep up with the progress of his science and art."

The Editors of "The Maryland Medical and Surgical Journal," in speaking of it, say—

"Its appearance in new dress is highly creditable to the publishers and editors. As a specimen of typography it is unrivalled by any of the numerous Journals which come to us. From the hasty examination of its contents, which as yet we have found time to bestow, we are inclined to think the "*matter*," unsurpassed by the accompanying "*dress*." * * *

"We cordially welcome this '*helper*,' in the cause of science, and feel that the present argues very favorably for the future numbers of our cotemporary. We hope its value will be fully appreciated by every friend of medical and dental literature."

It is thus noticed by the Editor of "The New York Lancet."

"Dental surgery is rapidly attaining that honorable position among the varied departments of science, to which it is entitled, although it is comparatively very recently that it has been prominently presented as a distinctive branch of scientific investigation. The above journal is published quarterly under the auspices of the 'American Society of Dental Surgeons,' an association which has lately been organized for the purpose of advancing the interests of this important branch of the profession, and exposing the shallow pretensions and impositions of quackery. The 'Journal' is conducted with great tact and talent, and appears to be well calculated to advance the great objects of the association, of which it is the organ. We will take an early opportunity of introducing our readers to a profitable acquaintance with several valuable articles in the numbers at present on our table."

To make it a medium of useful information by the publication of original and other papers connected with the theory and practice of dental surgery, and the republication of scarce and valuable works, is the wish and design of the association whose organ it is, and if there have been any so illiberal and narrow-minded as to suppose that its object was the subservance of any less noble purpose, we are happy to believe they are few in number, and found only among the ignorant and selfish. The sole object of the Journal, then, being the dissemination of such scientific truths as may be useful to the dental profession, we believe it will continue to be sustained. The present subscription to it is sufficient to defray the expenses of its publication, and the list of subscribers is steadily and constantly increasing; and since the commencement of the present volume there has been little cause of complaint on account of non-compliance with the terms of subscription. Most of the subscribers have paid cheerfully and promptly. There are, however, a few who are yet in arrears, and as the amount due from them is needed to complete the payment of the expense incurred for its last year's publication, we hope that none, after the receipt of the present number, will delay a day longer the payment of his subscription. And, while upon this subject, we would request of those who intend renewing their subscriptions, to forward the amount previously to the first of September, inasmuch as the first number of the next or third volume will be issued at that time.

